# Designing deep sequencing experiments: detecting structural variation and estimating transcript abundance

**DOI:** 10.1186/1471-2164-11-385

**Published:** 2010-06-18

**Authors:** Ali Bashir, Vikas Bansal, Vineet Bafna

**Affiliations:** 1 Dept. of Computer Science and Engineering, UC San Diego, La Jolla, CA, USA; 2 Scripps Genomic Medicine, Scripps Translational Science Institute, La Jolla CA 92037, USA

## Abstract

**Background:**

Massively parallel DNA sequencing technologies have enabled the sequencing of several individual human genomes. These technologies are also being used in novel ways for mRNA expression profiling, genome-wide discovery of transcription-factor binding sites, small RNA discovery, etc. The multitude of sequencing platforms, each with their unique characteristics, pose a number of design challenges, regarding the technology to be used and the depth of sequencing required for a particular sequencing application. Here we describe a number of analytical and empirical results to address design questions for two applications: detection of structural variations from paired-end sequencing and estimating mRNA transcript abundance.

**Results:**

For structural variation, our results provide explicit trade-offs between the detection and resolution of rearrangement breakpoints, and the optimal mix of paired-read insert lengths. Specifically, we prove that optimal detection and resolution of breakpoints is achieved using a mix of exactly two insert library lengths. Furthermore, we derive explicit formulae to determine these insert length combinations, enabling a 15% improvement in breakpoint detection at the same experimental cost. On empirical short read data, these predictions show good concordance with Illumina 200 bp and 2 Kbp insert length libraries. For transcriptome sequencing, we determine the sequencing depth needed to detect rare transcripts from a small pilot study. With only 1 Million reads, we derive corrections that enable almost perfect prediction of the underlying expression probability distribution, and use this to predict the sequencing depth required to detect low expressed genes with greater than 95% probability.

**Conclusions:**

Together, our results form a generic framework for many design considerations related to high-throughput sequencing. We provide software tools http://bix.ucsd.edu/projects/NGS-DesignTools to derive platform independent guidelines for designing sequencing experiments (amount of sequencing, choice of insert length, mix of libraries) for novel applications of next generation sequencing.

## Background

Massively parallel sequencing technologies provide precise digital readouts of both static (genomic) and dynamic (expression) cellular information. In genetic variation, whole genome sequencing uncovers a complete catalog of all types of variants including SNPs [1] and structural variations [2]. Transcript sequencing [3,4], small RNA sequencing and CHip-Seq [5] allow a measurement of dynamic cellular processes. These technologies provide unprecedented opportunities for genomics research but also pose significant new challenges in terms of making the optimal use of the sequencing throughput. The individual laboratory might not be equipped to provide correct, and cost-effective designs for the new experiments. By 'design', we refer to questions such as "How much sequencing needs to be done in order to reliably detect all structural variations in the sample to a resolution of 400 bp?" Confounding this further is the proliferation of a large number of sequencing technologies, including three widely used platforms, Roche/454 [6], Illumina [1] and ABI SOLiD [7], and others such as Pacific BioSciences [8] and Helicos [9,10]. These technologies offer the end-user a bewildering array of design-parameters, including cost per base, read-length, sequencing error rates, clone/insert lengths, etc. It is not straightforward to make a reasoned choice of technology and design-parameters in conducting a particular experiment. Likewise, the technology developers are faced with difficult choices on which parameters to improve in future development.

For any particular application, the goal of the researcher is to achieve the desired objective in a cost-effective manner. For example, in genome resequencing, the primary objective is the sensitive and accurate identification of various forms of sequence variants. Accurate SNP detection can be achieved even using short 36 bp Illumina reads [1]. However, for other applications such as de novo assembly of genomes, longer reads are significantly better than short reads [11]. RNA-seq is a novel application of sequencing to determine the expression levels of different mRNA transcripts in the cell [12]. However, the exponential variability in transcript expression levels poses new design questions regarding the required depth of sequencing to sample low abundance transcripts. Resolving such design questions can allow one to expand the scope of next-generation sequencing in novel directions. In this paper, we address and resolve some of the common design questions relating to structural variation and transcript profiling.

### Structural variation

**Figure 1:**
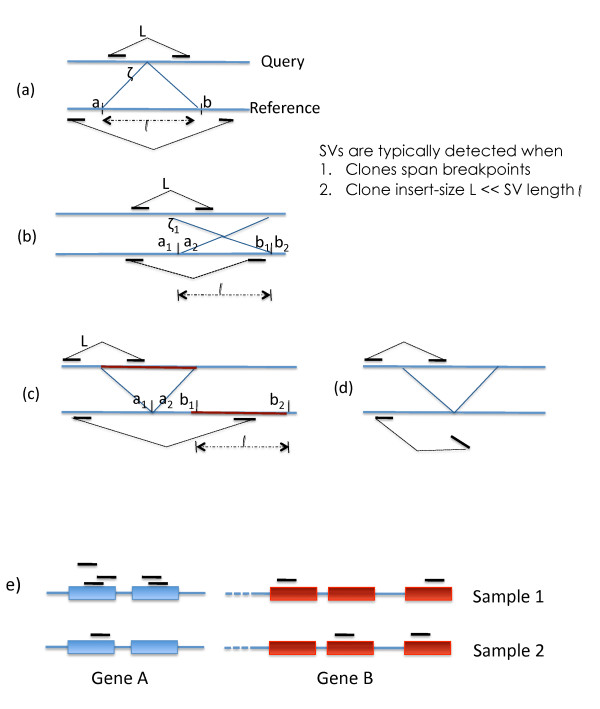
**Applications of next-generation sequencing**. Figure 1: (a) Structural variant (Deletion) detection using genomic PEM. The deletion brings disparate points (*a, b*) together to a fusion point in the query. (b) An inversion event is detected by detecting the two breakpoints (*a*_1_, *b*_1_), and (*a*_2_, *b*_2_) (c) An insertion caused by a translocation is detectable and has > 2 breakpoints. (d) Insertion of novel DNA causes dangling ends, and is harder to detect. (e) RNA-seq to detect gene expression. The number of reads mapped back to each gene indicate its relative abundance between and within a sample.

Structural variations (SVs) refer to events that rearrange a genome (*query*) relative to a reference genome [13] and include deletions, insertions, inversions and translocations of genomic regions. Paired-end Sequence Mapping (PEM) [14,15] represents a powerful approach to detect such events. In PEM, the ends of a large number of randomly selected inserts (clones) from the genome of an individual (query) are sequenced, and mapped to a reference genome. Inserts which map aberrantly to the reference genome in distance or orientation form an "invalid pair" and suggest an SV [14]. The general approach underlying PEM is illustrated in Fig. 1a-d. A number of recent informatics tools have been developed for the systematic detection of structural variation using the PEM framework [16,17].

#### Modeling SV detection

As detailed below, and in Figures 1a-d, SVs often involve the creation of *breakpoints*: a pair of coordinates (*a, b*) in the reference genome, that are brought together to a single location *ζ *in the query. Consider the *deletion *event in Figure 1a. A reference segment of length *l *= *b *- *a *+ 1 is absent in the query, relative to the reference. For the breakpoint (*a*, *b*) to be detected a paired-end insert must span *ζ*. Note that the insert-size is not fixed, but distributed tightly around a mean (*L *± *σ*). Deletion is confirmed if the breakpoint is spanned and *l *>>*σ*. Typically, *σ *<<*L *so we simply require that *l *>*L*, which is sufficient but not strictly necessary.

This approximation illustrates an important difference between 'algorithm design' for SV detection, and experiment design. Using a clever algorithm based on higher coverage, and variation in insert length (*σ*), it may be possible to detect smaller deletions (*σ *<*l *<*L*) as well. however, in deciding how much sequencing is done, we simply focus only on *l *>*L*. This simplification allows us to handle many different types of SV using identical design criteria. The similarity to other cases is described below.

The case of *inversion *is shown in Figure 1b. Here, two breakpoints, (*a*_1_, *b*_1_), and (*a*_2_, *b*_2_) are fused together in the query. Denote the length of the inversion SV as *l *= *b*_1 _- *a*_1 _= *b*_2 _- *a*_2_. The inversion is detected when both breakpoints are detected. As in the case of deletions, either breakpoint is detected when a shotgun insert of the query spans the corresponding fusion point, and has exactly one end-point inside the inversion. We enforce this by requiring that *L *<*l*, even though the condition is sufficient but not strictly necessary.

The case of *insertion *into a query sequence, relative to the reference, is slightly more complex, and can be broken into two sub-cases. In the first case (mediated by transpositions, or chromosomal translocations), a distal region *b*_1_, *b*_2 _of the reference genome is inserted at coordinate *a*_1_, creating at least 2 breakpoints ((*a*_1_, *b*_1_), and (*b*_2_, *a*_2_) in Figure 1c). Let *l *= *b*_2 _- *b*_1 _denote the length of the insertion SV. Again, SV detection depends upon the detection of 2 or more breakpoints. If on the other hand, the inserted sequence is not in the reference genome (Figure 1d), then detection is challenging, often involving a *de novo *assembly of the inserted region (see Bentley, 2009, Figure S21 [1]). We do not consider this case further. The case analysis above reveals the following common thread. An SV is characterized by its length *l*, and a collection of breakpoints. For an SV to be detected,

1. One or more of the SV breakpoints must be detected.

2. For each breakpoint:

(a) A shotgun insert must span the corresponding fusion point.

(b) The reads at the ends of the insert must map unambiguously to the ends.

(c) The insert-size must be dominated by the SV length (*l *>*L*).

This abstraction clarifies the design questions considerably. While the algorithmic questions must still deal with each SV separately, the design questions focus on breakpoint detection. We consider 2(b, c) first. For any choice of technology, and insert length, the distribution can be empirically computed by looking at concordantly mapped reads. Using this distribution, we can compute the probability of a randomly picked insert having a specific size.

Consider a typical experiment for SV detection. The researcher would like to detect a large fraction of all SVs of length ≥ *l*, with high confidence (≥ 1 - *ε*). They must choose (a) a specific instrument technology; (b) insert-size(s) from the ones available; (c) read-length, and (d) the amount of sequencing. First, the researcher must choose a technology and insert-size constraint, where(1)

The choice of a specific read-length is somewhat less important, but the reads must be long and accurate enough to map unambiguously. We model both points by introducing a parameter *f*, referring to the fraction of reads that map unambiguously. Therefore if *N *inserts must map unambiguously to satisfy design constraints, then *N/f *inserts need to be sequenced, on the average. In the remainder, we limit the discussion to detecting breakpoints, considering only the technologies and insert sizes that satisfy the size-constraint (1); and, we assume a mapping parameter *f *to scale the answers. The issue now is to choose from available insert-sizes, and second, to determine the amount of sequencing. In this paper, we formulate, and resolve design issue 2(a) as:

• *Given a choice of insert-sizes, and parameter ε, compute the amount of sequencing needed to detect *1 - *ε of all breakpoints in the query genome*.

We address the questions of breakpoint detection conjunction with the related notion of breakpoint *resolution*. With most technologies, a breakpoint detected as a pair of regions ([*a*_1_, *a*_2_], [*b*_1_, *b*_2_]), such that *a *∈ [*a*_1_, *a*_2_], and *b *∈ [*b*_1_, *b*_2_]. The resolution, defined by |*a*_2 _- *a*_1_| + |*b*_2 _- *b*_1_| refers to the uncertainty in determining (*a, b*). Good resolution is critical elucidating the phenotypic impact of the variation In an earlier work, we described the use of tightly re solved breakpoints in detecting gene fusion events cancer [18]. This framework was extended to form general geometric approach for detecting structural variants [16]. We reformulate and resolve the question

• *Given a choice of insert-sizes, and parameters ε, s, compute the amount of sequencing needed to detect *1 - *ε of all breakpoints in the query genome to a resolution of ≤ s bp*.

Intuitively, the likelihood of detection would be maximized by choosing the largest available insert-size. However, the longer insert-sizes increase the uncertainty in resolving the breakpoint. One result of our paper is an explicit trade-off between detection and resolution. We also derive a formula that computes the probability of resolving a breakpoint to within '*s*' base-pairs, given a fixed number of shotgun reads from a specific paired-end sequencing technology. Another result of our paper is that it is advantageous to use a mix of insert-sizes. For example, we can show that only 1.5× mapped sequence coverage of the human genome using Illumina (Solexa) can help resolve almost 90% of the breakpoints to within 200 bp using a mix of inserts. All other parameters being equal, we show that the best resolution of a structural variant comes from using exactly two possible insert-lengths: one that is as close as possible to the desired resolution, and one that is as long as technologically possible (with reasonable quality).

In summary, the researcher can use our formulae in designing his experiment to (a) select appropriate insert-sizes; (b), the optimum amount of sequencing for each insert library. A web-based tool based on the above is available.

### Transcript sequencing

Transcript sequencing is a direct approach for measuring abundance, and variations involving splicing, and SV mediated gene disruptions, and fusions [3]. In most transcript sequencing methods, RNA is fragmented, and converted into cDNA, which is subsequently sequenced and mapped back to a reference [12]. This protocol has shown great promise in detecting aberrant splice forms and SVs that lead to gene disruptions, and fusions [4].

Often, transcript sequencing is used for gene expression profiling. See Figure 1e. The significant difference in sampled reads (5 to 1) between Samples 1 and 2 suggests that gene A's expression level has changed between the two samples. In measuring relative abundance, RNAseq mimics older technologies like microarrays. However, sequencing stands alone in being able to compute relative abundance between two distinct transcripts. In sample 1, the difference in read coverage between genes A and B suggest that A is more than twice as abundant as B (assuming A and B are approximately the same length).

Let *x*_*t *_denote the *true-expression *of transcript *t*, defined as the number of copies of *t *in the sample. Additionally, the transcript is broken into a number of pieces, roughly proportional to its length, *l*_*t*_. Therefore, we assume that transcript *t *yields ∝ *l*_*t*_*x*_*t *_copies in the sample [3]. This contrasts with earlier technologies like EST sequencing, which were biased towards the 3' (or 5') end. Let *a*_*t *_denote the number of sequences sampled from *x*_*t*_. We denote the *normalized-expression *for *t *(likelihood of a randomly sampled read coming from *t*) by

A typical design question for transcript sequencing is to determine the amount of sequencing required to sample a given fraction (Say, 90%) of the expressed transcripts. The question is particularly difficult to answer because different transcripts have vastly different normalized-expression values. Using empirical and analytical observations, we show that the p.d.f of the normalized-expression can be computed using a small sample. Therefore, a researcher can start with an initial sequencing run (< 500 K reads), and use the mapping data to compute the additional amount of sequencing needed. Formally, we resolve the following:

• *Given transcript mappings from a small sample of sequences, and parameter ε, compute the amount of additional sequencing needed to detect *1 - *ε of all expressed transcripts*.

Our results are based on novel extrapolation for the low abundance genes that are not accurately represented in the sample. They allow the researchers to efficiently allocate resources for large RNA sequencing studies. This is particularly relevant when many related samples are being sequenced and one needs to assess the trade-offs between sequencing depth and sample coverage.

## Results and Discussion

### Structural Variation

As discussed in the introduction, we can limit the question of SV detection to detection of SV breakpoints. Let breakpoint (*a, b*) in the reference genome fuse to a single point *ζ *in the query genome. Let *P*_*ζ*_, denote the probability that an arbitrary breakpoint is detected. Our goal is to derive an expression for *P*_*ζ*_, given a certain amount of sequencing.

Direct application of breakpoint formulae requires that one selects from insert-sizes that are smaller than the desired SV length. In the following, we work with available inserts, where the mean insert-size ranges from *L *= 200 bp to *L *= 10 Kbp. Therefore, a result that says *P*_*ζ *_= 0.9 can be interpreted to mean that 90% of all breakpoints from SVs of length significantly larger than *L *Kbp can be detected. These specific values are chosen for illustration purposes only. Identical results apply for smaller or larger SVs, except that we would be limited to choosing from appropriate insert-sizes. All analytical results are derived assuming a fixed value for *L*. However, all results on real data use the natural variation in insert-size, and show excellent concordance with the analytical results.

#### Detection-Resolution trade-off

Consider *N *inserts with fixed insert-size *L *sampled at random and end-sequenced. For a genome of length *G*, the clonal coverage *c *= *NL/G*, describes the expected number of inserts spanning *ζ*. A breakpoint is detected exactly when at least one insert spans *ζ*. Therefore, *P*_*ζ*_, the probability of detecting an arbitrary breakpoint, is given by the Clarke-Carbon Formula [19,20].(2)

Equation 2 demonstrates the effect of *L *and *N*. Larger values *L *(among allowable insert-sizes), or the amount of sequencing *N *improve the probability of detection. However, the greater insert length also creates a greater uncertainty in the location of *ζ*. Define *resolution-ambiguity *as the size of the region *θ *(denoted by |*θ*|) in which *ζ *is constrained to lie. Order the inserts spanning *ζ *by their right endpoint. Let *A *be the distance of the right end point of the leftmost insert to the right of *ζ*. Then,

We show (see METHODS) that

Using symmetry arguments,(3)

Equations 2, and 3 provide an SV detection versus resolution trade-off. For a fixed number of sequences *N*, increasing *L *increases the probability of detection, but also increases the resolution-ambiguity. The effect decreases for large *N*. To validate this using experimental data, we used the publicly available Illumina generated human reference sequence from NA18507, a Yoruban male [1]. Using the complete data, we computed a set of "true breakpoints" from SVs of length ≥ 2000 (see METHODS).

**Figure 2:**
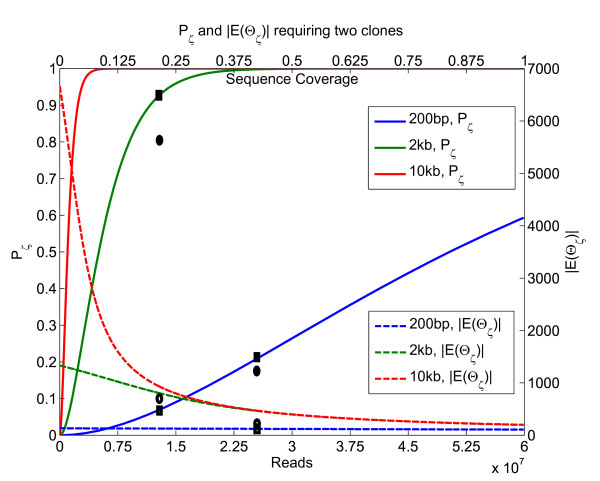
**Detection-resolution trade-off**. Figure 2: The detection probability, *P*_*ζ *_(left-axis), increases with increased sequencing (*N*), as well as insert-length (*L*). The expected resolution-ambiguity, |*E*(Θ_*ζ*_)| (right-axis), increases with increasing insert-length *L*. The bottom x-axis shows total number of reads (*N*) while the top x-axis shows the corresponding sequence coverage (with 50 bp paired reads relative to the human genome). □' and '●' correspond to the expected and observed values, respectively, for specific values of *N *and *L*.

Next, we collected all inserts with mean insert-size either 200 bp, or 2000 bp. Choosing the number of mapped reads as a parameter *N*, we collected random sub-sets of *N *paired-reads, and computed the fraction of true breakpoints detected as well as the expected resolution (see METHODS). Figure 2 illustrates the trade-off between detection and resolution. The plotted-lines correspond to theoretical predictions which do not use variance in insert-sizes. The dark ovals show the experimentally observed values for detection and resolution, which can be compared against the corresponding theoretical values (squares).

Nevertheless, current sequencing capability allows us to detect and resolve a large fraction of breakpoints. For example, with an Illumina run with 2 Kbp inserts and 25 × 10^6 ^mappable reads one could detect nearly 100% of breakpoints with an average resolution-ambiguity of less than 500 bp.

#### Mixing insert lengths

Many of the next generation sequencing technologies offer a variety of insert lengths. For example, the ABI SOLiD technology claims a variety of insert lengths ranging from 600 bp to about 10000 bp [21]. Given the trade-off between detection and resolution, we next asked if using a mix of insert lengths could help with detection *and *resolution. To address this, we first derived bounds on the probability of resolving a breakpoint to a desired level of resolution using a mix of two insert lengths. Suppose we generate *N*_1_, *N*_2 _reads, respectively from insert libraries of lengths *L*_1_, *L*_2_. Then, for an arbitrary *s *(see METHODS)(4)

Note that the resolution-ambiguity |Θ| ≤ *L*_1_, or |Θ| = *L*_2 _can be obtained using single insert libraries, but the likelihood of resolving between *L*_1 _and *L*_2 _is optimized by using an appropriate mix of the two libraries. Analogous equations can be derived when two overlapping inserts or more are required to detect a breakpoint.

**Figure 3:**
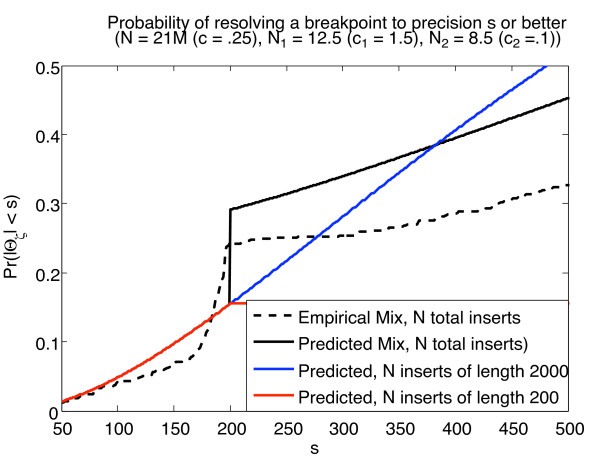
**Combination of insert lengths for breakpoint detection**. Figure 3: The solid lines indicated the theoretically predicted probabilities as described in METHODS. The dotted line indicates an empirical mix, using 4 lanes each from 200 and 2000 base pair insert runs. *N *(*c*) correspond to the combined number of reads (sequence coverage) uniquely mapped at both ends.

Figure 3 illustrates this principle using publicly available Illumina generated human reference sequence from NA18507, a Yoruban male [1], assuming one had chosen to split a single run (flow cell) between 2 insert-sizes. As described earlier, we first used the complete data to compute a set of "true breakpoints" from SVs of length ≥ 2000 (see METHODS).

Next, we collected all inserts with mean insert-size either 200 bp, or 2000 bp. For a fixed amount of sequencing, we confirmed the theoretically predicted boost in probability of detecting a breakpoint to within a resolution-ambiguity of 200 bp. The results are in Figure 3. The probability is doubled from 0.15 to over 0.29 using a mix of insert libraries. Similar results are obtained for other sequencing studies, such as an ABI SOLiD sequencing with 600 and 2700 length libraries (data not shown). In a further extension of the analysis, we show that to maximize the likelihood of resolving breakpoints to *s*bp, we need only two libraries-one with insert-length *s*, and the other as large as possible (see METHODS). A restatement of these results can be found in Additional file 1. We note that only 1.5× mapped sequence coverage of the human genome using Illumina (Solexa) can help resolve almost 90% of the breakpoints to within 200 bp using a mix of inserts. Similar results were obtained when applied to runs from the ABI SOLiD system [21].

While our analytical results treat the insert sizes as fixed, empirical data very closely approximates the theoretical curve (Figure 3, dotted lines). Though the theoretical model performs better (mostly due to mapping variation resulting from repeat-like genomic regions), the magnitude of the 'boost' at 200 bp is maintained. The concordance between theoretical and experimental results shows the limited effect of insert-length variation.

It is useful to revisit the case of SVs with very small lengths. Mechanisms such as non-homologous end-joining (NHEJ), often gives rise to small insertions and deletions [2], that are valuable as genetic markers. If the event size is smaller than the variance in available insert-size, the event will not be detected by paired end mapping (in the case of deletions and insertions). In these situations, detection is improved by longer reads (such as those available in Roche-454). If single reads are used to detect the fusion point, then there is no ambiguity in resolution. In that case, the design question becomes simple, and the desired number of reads can be computed using the Clark-Carbon formula, and scaled using the mapping parameter *f*.

### Transcript sequencing

As transcripts have variable expression, the amount of sequencing needed to detect a transcript is variable. A key design issue is to determine if sufficient sequencing has been performed to sample all transcripts at a certain expression level. For example, in large patient surveys one needs to identify the number of samples that can be sequenced at minimal cost, while ensuring detection of genes at a desired expression level. Similarly, when evaluating a given sample it is important to know whether the required sequencing depth has been reached, or if more sequencing is necessary to detect a given transcript, isoform, or fusion gene. We show here that a relatively low level of transcriptomic sequencing has sufficient information regarding the variability of expression that it can be used to compute the likelihood of a specific transcript being sampled.

While deep sequencing is required to accurately estimate the normalized expression, *ν*_*t*_, for each transcript, *t*, a more modest level of sequencing allows us to estimate the distribution of *ν *values among all transcripts. Formally, define a p.d.f *f*(*ν*) for a randomly sampled transcript to have normalized-expression *ν*. Consider a transcript sequencing experiment with *N *reads. If we could estimate *ν*_*t*_, then

Instead, we propose to use the estimate of *f *to make predictions about sampling transcripts.(5)

We tested the predictive accuracy of Eq. 5 using data from Marioni et al. [3]. An empirical p.d.f was derived (see METHODS) from the total sequence used in each of two tissue studies (kidney and liver, ~35 × 10^6 ^reads each). Additional file 2a shows the similarity between the empirical distribution of normalized-expression values between the two studies.

**Figure 4:**
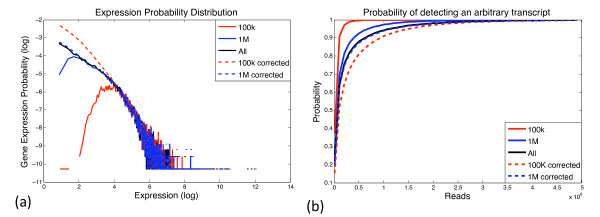
**Transcript abundance distribution predicted from sampled reads**. Figure 4: (a) Each point on a curve (solid-line) corresponds to the number of transcripts (Y-axis) that had a specific normalized count (X-axis). Note that the number of transcripts small counts relative to coverage drops sharply. For example, the curve for 100K drops at log(*X*) ≤ 5. This undersampling is corrected by the dotted lines. This correction enables the computation of (b), the probability of detecting an arbitrary transcript. The solid lines correspond to predictions made of the empirical (or simulated empirical) distribution. The dotted lines correspond to corrected values from regression (see METHODS). Note the high fit that is obtained after correction, with only 100,000 reads.

We next asked if *f *could be accurately estimated using a lower sequencing depth. If so, this lower level of sequencing can be used to compute the depth of sequencing required to adequately sample all of the transcripts. To test this, smaller sequence-subsets (100 K, and 1 M) were generated by sampling from the complete set. Expression distributions were computed from each subset as shown in Figure 4a. These were then used to compute the probability of transcript detection. Figure 4(b) plots a *detection-curve*, described as the probability of detecting a transcript from the liver sample as a function of its normalized abundance. While predictions made with smaller samples (blue, red solid lines) roughly track the true detection-curve (black line), there is significant bias as low abundance reads are not accurately sampled (Additional file 3).

Previous work has indicated that gene expression distributions typically follow a power-law [22,23]. Nacher et al. extended this idea, accounting for stochastic noise to provide better fits for low expressed genes [24]. We created a novel regression based strategy (METHODS) to correct for the bias, by fitting a power-law to high-expressed genes and using the simplified variant of models proposed by Nacher et al, to accurately approximate genes with low expression levels. The corrected curves (blue, red dotted lines) track the true estimates closely, even when using a sparse set of 100 *K *reads. With 1 million reads, > 90% of the total observed transcripts were sampled. In this data *f *is well-conserved across samples (as seen in kidney and liver, Additional file 2a). For example, the expression p.d.f. for kidney can be used to roughly predict the probability of detection for liver (Additional file 2b). This implies that *f *may not need to be re-estimated independently for related samples.

## Conclusions

We present a number of analytic and empirical results on the design of sequencing experiments for uncovering genetic variation. Our study provides a systematic explanation for empirical observations relating to the amount of sequencing, and the choice of technologies. The theoretical analysis is not without caveats, which are discussed below. Nevertheless, the concordance with empirical data illustrates the applicability of our methods. Some of the results, while not counter-intuitive, provide additional insight. For example, we show that the best design for detecting SV to within 's' bp demands the choice of exactly two insert-lengths, one close to *s*, and the other as large as possible. We explicate the trade-offs between detection and resolution, and provide a method for computing the probability of SV detection as well as the expected resolution-ambiguity for a variety of technology and parameter choices.

Many additional confounding design issues that can be modeled in the context of structural variation. Different technologies have different error rates. This is corrected by introducing a mapping-rate parameter *f*, defined as the fraction of reads that are mapped unambiguously to the reference. Replacing the number of reads *N *by *fN *helps correct somewhat for sequencing errors. New methods have been suggested for dealing with complex scenarios in which it is difficult or impossible to map reads uniquely, such as within recent segmental duplications, using hill climbing [25] or parsimony [26] based approaches which try to minimize the number of observed structural variants. Chimerisms in insert-lengths can be controlled by demanding the use of multiple overlapping inserts. We have extended most analyses to requiring two or more inserts (see METHODS).

An important simplification in our analysis is to treat insert-length as constant. However, choosing a distribution on the insert-length does not influence the expected resolution-ambiguity, only its variance. The variance is important for measuring smaller structural variations. Therefore, experiments that aim to detect small structural variations are constrained to using technologies in which the insert-length variation is significantly smaller than the size of the SV itself. The available technologies are constantly reducing the variance in insert-lengths through better library preparation strategies, which might allow the use of larger insert-lengths in the future.

For transcript sequencing, we address the important question of depth of sequencing, given the large variation in transcript abundance. Our results suggest that estimating the distribution of normalized expression values with modest amounts of sequencing can help address design questions for transcript sequencing, even when the transcript abundance varies over many orders of magnitude. This approach has a number of caveats, for example, it assumes unbiased sampling of transcripts. Current library preparations have been shown to have biases (such as 3' and 5' depletion) [12] as well as biases towards specific RNAs (specifically small RNAs) within a platform [27]. Additionally, though our results indicate a very good empirical fit on human samples, the assumption of a power-law, or other distribution, may not fit all samples. A number of outstanding questions remain, such as the detection of splicing events, and the resolution of breakpoints. While transcript sequencing is a quick way to detect breakpoints, the location of the breakpoint is confounded by trans-splicing. The issues relating to design can be better resolved only after methods are discovered to resolve breakpoints and predict splicing events based on transcriptome sequencing.

We do not address some important applications of next generation sequencing technologies: the detection of rare (and common) sequence variants in re-sequencing studies. Given the relatively high error rates for some of these technologies, reliable and accurate detection of sequence variants (SNPs) is a challenging problem, and general design principles that would be applicable to all technologies will be addressed in future study. The design of sequencing for 'dark-region' identification (i.e. DNA inserts on the sampled genome that are not in the reference) is not addressed. Lastly, there are practical sample preparation issues which demand consideration. Longer insert-lengths consume more sample for equivalent amount of sequencing. Therefore, if the sample is limited (as in tumors), the best design should also seek to optimize a 'sample-cost' versus detection trade-off.

Technological developments all point to the rapid deployment of personalized genomic sequencing. As large populations of individuals are sequenced, and the sequence is analyzed for a variety of applications, design issues relating to the amount of sequencing, the choice of technology, and the choice of technological parameters become paramount. Our paper helps resolve some of these questions. As current technologies mature and new technologies arise it will be critical to further develop a framework to maximize study efficacy.

## Methods

### Breakpoint Resolution

The insert coverage is given by *c *= *N L/G *where *N *is the number of inserts. A breakpoint (*a, b*) in the reference genome corresponds to a fusion point *ζ *in the query genome where the coordinates *a, b *come together. Let *ζ *be covered by at least one insert, and let *A *be the distance of the right end point of the leftmost insert from *ζ*.

Therefore,

Using symmetry arguments,

Requiring coverage by multiple (≥ 2) inserts,

### Simulation

A set of "true" breakpoints were chosen by mapping Illumina reads for individual NA18507 (obtained from the NCBI short read trace archive) to build 36.1 of the human genome. ELAND alignment tool, where each end mapped separately to detect SVs. Insert libraries were mapped until > 100× insert coverage was reached, in order to obtain a candidate set. To avoid systematic errors within a library (and over-fitting of the test data) at least three distinct libraries were required to span a breakpoint for it to be considered a "true breakpoint". All SV events greater than 2 Kbp were selected to be the final set is considered to be the TRUEBREAKPOINTSET.

To test the theoretical predictions, a 200 bp and a 2 Kbp library were selected at random. For parameter *N*, we randomly picked *N *paired-reads in which both ends mapped uniquely to the genome. A true breakpoint was considered to be detected if at least two inserts spanned it. Thus, the fraction of true breakpoints detected was empirically computed. These numbers were compared against theoretical predictions, obtained using Eq. 2,3 respectively. The resolution |Θ_*ζ*_| for each detected break-point was computed as follows: for each paired-read that spanned a breakpoint, let *x*_*l *_denote the distance of its left endpoint from the left end of the right-most clone; let *x*_*r *_denote the distance of its right end-point from the left most clone. Then, the resolution is given by *L *- (*x*_*l *_+ *x*_*r*_). |Θ_*ζ*_| was obtained by taking the mean (Θ_*ζ*_) of all overlapping paired-reads. The fraction of "true" breakpoints detected (at least 2 inserts spanning the event) and resolved by these libraries is shown in Figure 3, as a function of *N*.

### Mixing insert lengths

Consider the case where we have two different insert-lengths *L*_1 _and *L*_2 _where *L*_2 _>*L*_1 _w.l.o.g. Denote the coverages of the insert libraries as *c*_1 _and *c*_2_. Let *c *= *c*_1 _+ *c*_2_*L*_1_/*L*_2_

Next, we compute the probability of resolving a breakpoint to within 's' bp. We have 3 cases: i) *s *<*L*_1_; ii) *L*_1 _≤ *s *<*L*_2_; and, iii) *s *> = *L*_2_. For *s *<*L*_1_, we extend the analysis of [18], where we showed that . Denoting *N *= *N*_1 _+ *N*_2_,

Note that the results are independent of insert-lengths (or, in fact, whether or not a mix of inserts is being used). However, for the case *L*_1 _≤ *s *<*L*_2_, we have to consider the event of an *L*_1 _insert spanning the breakpoint *or *the event of two *L*_2 _inserts spanning *ζ *with no *L*_1 _inserts spanning *ζ *Therefore,

The case when *s *>*L*_2 _can be modeled by a single library with *c *= (*N*_1_*L*_1 _+ *N*_2_*L*_2_)/*G*.

The equations can be modified to require that at least 2 inserts overlap a breakpoint. Case (i) is unchanged, as it requires 2 inserts. Likewise for the second term in case (ii). We constrain case (ii) to require 2 or more inserts for the first term.

For case (iii), we can extend the generic cluster coverage case.

### Proof of Optimality of Two Insert Design

We show that it is sufficient to consider exactly two insert lengths for resolving a breakpoint to within 's' bp. We show first that for a given *s *and *N*, and a collection of insert-lengths, *Pr*(|Θ_*ζ*_| = *s*), is maximized using a mixture of ≤ 2 insert lengths.

Assume to the contrary that an optimal mix requires ≥ 3 distinct insert-lengths. This implies that for some insert length *L'*, *L *≠ *s*, and *L *≠ *L*_*M*_, where *L*_*M *_is the maximum available insert-length. In other words, either a) *L' *<*s*, or, b) *s *<*L' *<*L*_*M*_. We consider each case in turn.

*L' *<*s*: From earlier discussion, the contribution of the inserts with length *L' *to *Pr*(|Θ| ≤ *s*) is proportional to coverage (*c*_1_). Replacing inserts of length *L' *with inserts of length *s *will increase coverage without changing *N*, contradicting optimality.

*s *<*L' *<*L*_*M*_: Once again, for inserts larger than the desired resolution-ambiguity *s*, their contribution to *Pr*(|Θ| ≤ *s*) is completely dependent on coverage. Replacing by a insert of length *L*_*M *_improves the resolution probability, a contradiction.

An immediate corollary is that the optimal design consists of a mix of two insert lengths, *s *and *L*_*M*_. The mix of the two libraries (the ratio *N*_1_/*N*_2 _s.t. *N*_1 _+ *N*_2 _= *N *is fixed) only needs to be optimized for Case (ii).

We compute the optimal mix empirically by iterating over *N*_1 _∈ [0, *N*].

### Simulation for mix of inserts

The set of breakpoints, and method for computing mean size of Θ_*ζ*_, followed that of the previous simulation. A single 2 Kbp and 200 Kbp library were analyzed, using 4 lanes from each corresponding flow cell. Clusters of invalid pairs were generated by combining the two reduced libraries.

### Transcript Sequencing

Mapped RNA-seq data, generated by Marioni, et al. [3], was obtained from http://giladlab.uchicago.edu/data.html. The genomic mappings were converted to a list of overlapping exons in Refseq. For each transcript, a count of the number of reads sampling it was generated. This enabled the estimation of *ν*_*t *_which was calculated as described earlier. To obtain smaller data sets, random sampling of the reads was performed and *ν*_*t *_was re-calculated.

The sample is used to estimate the p.d.f of normalized abundance values, and is shown in Additional file 2. It can be observed that each sample of *r *reads is accurate for highly expressed genes (normalized expression > 1/*r*). Below 1/*r*, the chance of sampling a gene is low, and so the p.d.f cannot be estimated accurately. It has been shown empirically that most tissues follow a power law distribution [22,23].

Figure 4a shows a plot of log *f*(*ν*) vs. log(*ν*). Performing a regression analysis on the line reveals the slope *α*, and the intercept log(*β*).

Nacher et al. suggested a stochastic model of gene expression which, in practice, provides a better fit to gene expression data. They provide the equation:(6)

Where *δ*_*D *_is a noise parameter (relating to decay of RNA molecules) and *N *is a normalization constant [24]. Note, that this equation is approximated by the power law  at high values of *ν*.

Generating the fit requires two important steps: fitting a power law at high gene expression and identification of a "reliable point". Note that "high gene expression" can be maintained for all samples (in our simulations we used  of overall expression). Performing a regression on gene expression values above this threshold provides *δ*_*D*_. Intuitively, the reliable point can be identified independently for each distribution by determining the point of inflection of the graph log(*f*(*ν*)) vs *log*(*ν*); the set of points immediately downstream of the inflection are used to fit Equation 6. One can accurately determine a "reliable point", *ν*_*r*_, by computing the gene expression value at which there is a 95% probability of detecting a transcript, , where *r *is the number of reads. The corrected p.d.f. utilizes the empirically generated p.d.f after this reliable point, and the theoretical p.d.f before then. It is important to note that the empirical p.d.f. derived using all reads implies that there is a drop off in abundance for very low abundance genes, which the fitting procedure would over-predict. However, this could be an artifact of incomplete sampling and a regression of the full data may provide a better estimate.

## Authors' contributions

A.B., V.Bansal, and V. Bafna all conceived the study, designed the experiments, and wrote the manuscript. A.B. and V. Bansal implemented all of the software related to the study.

## Supplementary Material

Additional file 1 **The statistics obtained in this research have been implemented in tools, available via web-service and download at **http://bix.ucsd.edu/projects/NGS-DesignTools. Figure 3: An optimal mix of inserts (200 bp and 2 kb) improves the probability of resolving breakpoints to a given precision (200 bp). The probability was computed for an experimentally computed set of "true breakpoints" for SVs of length greater than 2000 bp. The bottom x-axis shows total number of reads while the top x-axis shows the corresponding sequence coverage (with 50 bp paired reads relative to the human genome). Different ratios consistently outperform/underperform one another - a single insert size consistently underperforms any ratio of mixed insert sizes. Click here for file

Additional file 2 **The statistics obtained in this research have been implemented in tools, available via web-service and download at **http://bix.ucsd.edu/projects/NGS-DesignTools. Figure 1: (a) Distribution of normalized expression from two transcript sequencing experiments. (a) Histogram of *ν *in two separate samples. For clarity, the bins are log distributed and the y-axis represents the fraction of total reads. (b) Fraction of detected transcripts from kidney RNA-seq at different sequencing depths. The expected distribution from a different tissue (liver) tracks, but typically over predicts probability of detection at higher sequencing depths. Click here for file

Additional file 3 **The statistics obtained in this research have been implemented in tools, available via web-service and download at **http://bix.ucsd.edu/projects/NGS-DesignTools. Figure 2: Estimating the p.d.f of normalized gene expression values. Note that all samples agree except at low levels of detection, where there are insufficient reads. Thus, the 100 K (10^5^) reads sample can only estimate the p.d.f accurately after a normalized expression value of 10^-5^. Click here for file
