# Supplementary figures and images for: Designing deep sequencing experiments: detecting structural variation and estimating transcript abundance
Source: BMC Genomics. 2010 Jun 18;11:385. doi: 10.1186/1471-2164-11-385 (PMC3091630; doi:10.1186/1471-2164-11-385)

Probability of detecting a breakpoint to 200 bp or better 200 and 2kb insert lengths

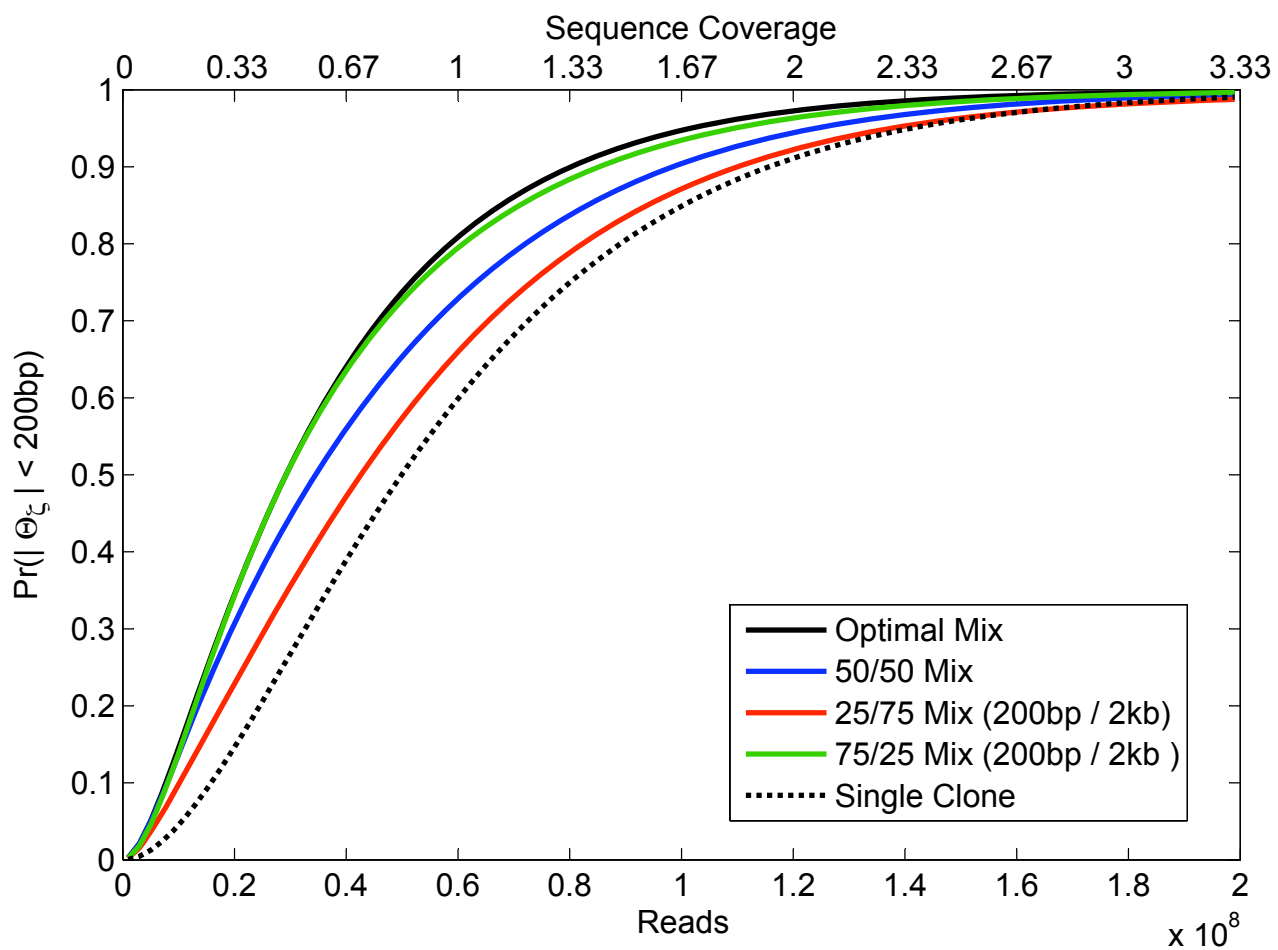

Supplement: Additional file 1 — The statistics obtained in this research have been implemented in tools, available via web-service and download at http://bix.ucsd.edu/projects/NGS-DesignTools. Figure 3: An optimal mix of inserts (200 bp and 2 kb) improves the probability of resolving breakpoints to a given precision (200 bp). The probability was computed for an experimentally computed set of "true breakpoints" for SVs of length greater than 2000 bp. The bottom x-axis shows total number of reads while the top x-axis shows the corresponding sequence coverage (with 50 bp paired reads relative to the human genome). Different ratios consistently outperform/underperform one another - a single insert size consistently underperforms any ratio of mixed insert sizes. [file 1471-2164-11-385-S1.PDF]

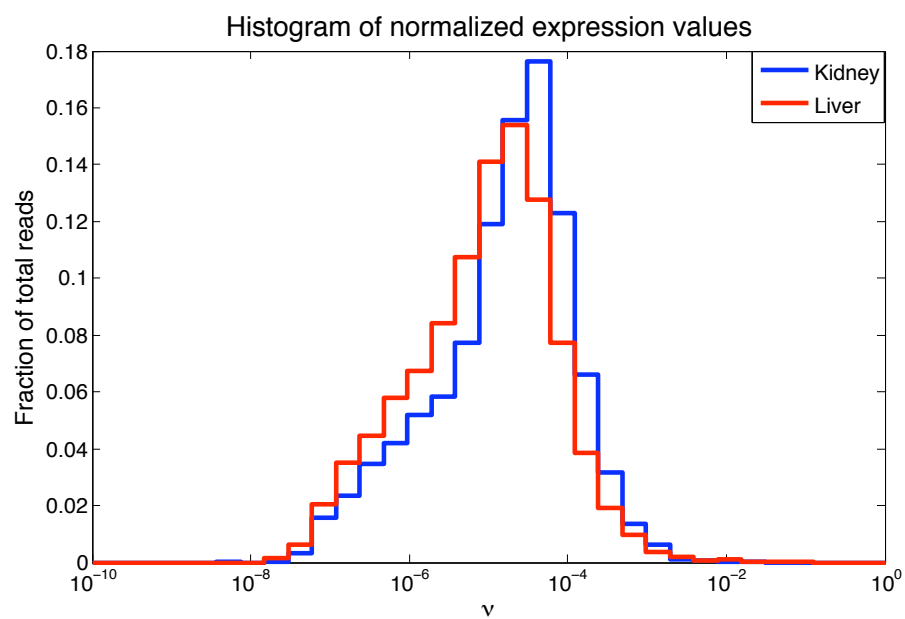

(a)

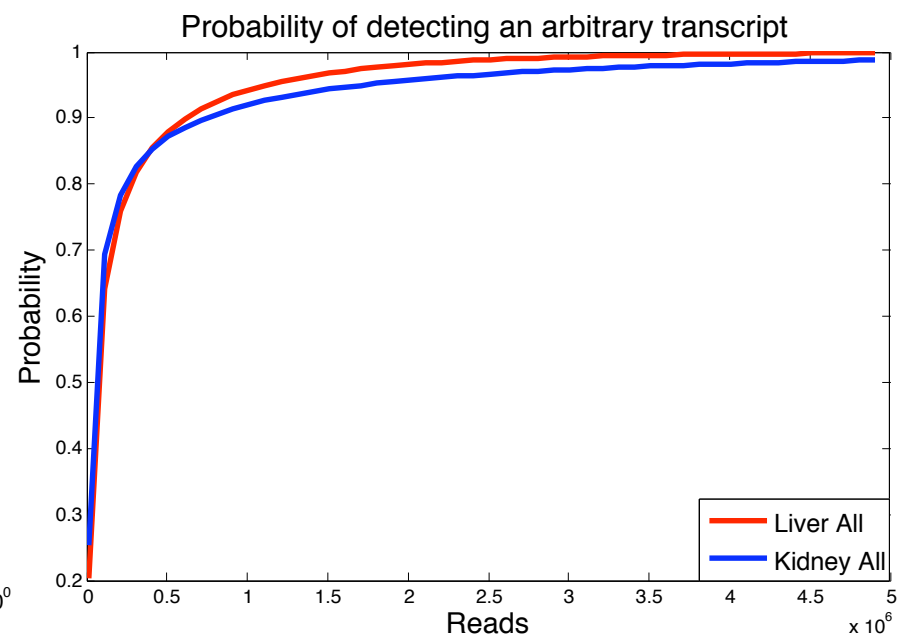

(b)

Supplement: Additional file 2 — The statistics obtained in this research have been implemented in tools, available via web-service and download at http://bix.ucsd.edu/projects/NGS-DesignTools. Figure 1: (a) Distribution of normalized expression from two transcript sequencing experiments. (a) Histogram of ν in two separate samples. For clarity, the bins are log distributed and the y-axis represents the fraction of total reads. (b) Fraction of detected transcripts from kidney RNA-seq at different sequencing depths. The expected distribution from a different tissue (liver) tracks, but typically over predicts probability of detection at higher sequencing depths. [file 1471-2164-11-385-S2.PDF]

Raw Expression Probability Distribution

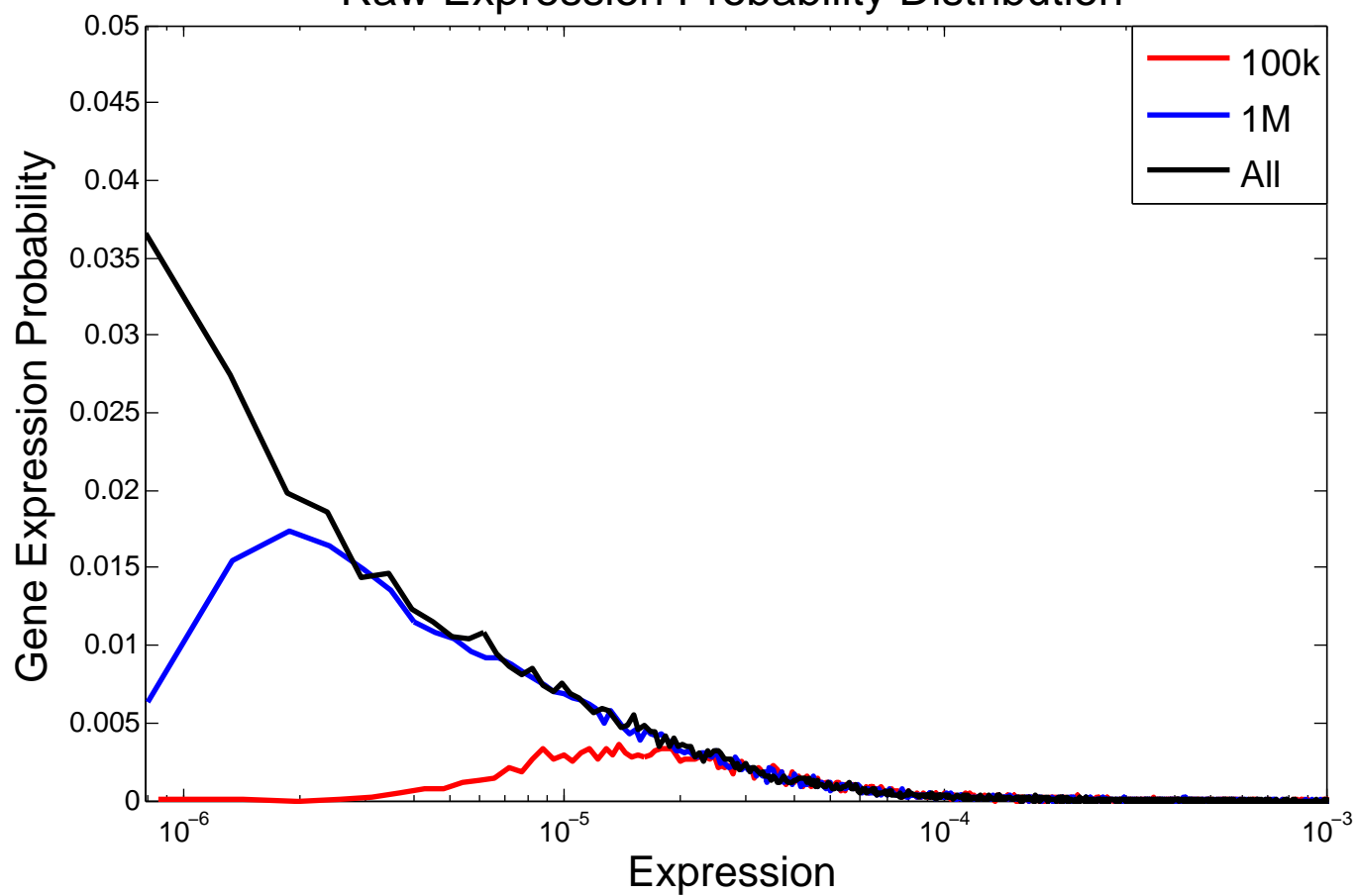

Supplement: Additional file 3 — The statistics obtained in this research have been implemented in tools, available via web-service and download at http://bix.ucsd.edu/projects/NGS-DesignTools. Figure 2: Estimating the p.d.f of normalized gene expression values. Note that all samples agree except at low levels of detection, where there are insufficient reads. Thus, the 100 K (105) reads sample can only estimate the p.d.f accurately after a normalized expression value of 10-5. [file 1471-2164-11-385-S3.PDF]
